# Chimpanzees and children are curious about social interactions

**DOI:** 10.1098/rspb.2024.2242

**Published:** 2025-06-04

**Authors:** Laura Simone Lewis, Oded Ritov, Esther Herrmann, Rachna B. Reddy, Alejandro Sanchez-Amaro, Alison Gopnik, Jan M. Engelmann

**Affiliations:** ^1^ Departments of Psychological and Brain Sciences and Anthropology, University of California, Santa Barbara, CA, USA; ^2^ Department of Psychology, University of California, Berkeley, CA, USA; ^3^ School of Psychology, Sport and Health Sciences, University of Portsmouth, United Kingdom; ^4^ Department of Anthropology, The University of Utah, USA; ^5^ Department of Psychology, University of Stirling, United Kingdom

**Keywords:** social curiosity, cognitive development, comparative psychology, primates, chimpanzees

## Abstract

Curiosity is adaptive, enhances learning, and reduces uncertainty. Social curiosity is defined as the motivation to gain information about the actions, relationships, and psychology of others. Little is known about the developmental and evolutionary roots of social curiosity. Here, across three comparative studies, we investigate whether chimpanzees (*n* = 27) and young children (4–6 years old, *n* = 94) show particular interest in social interactions among third parties. Chimpanzees and children preferred to watch videos of social interactions compared with videos of a single conspecific (Experiment 1) and young children and male chimpanzees even paid a material cost to gain social information (Experiment 2). Finally, our results show that boys become more curious about negative social interactions whereas girls become more curious about positive social interactions as they develop, while chimpanzees demonstrated no preference for negative versus positive social interactions (Experiment 3). Taken together, these findings suggest that social curiosity emerges early in human ontogeny and is shared with one of our two closest living relatives, the chimpanzees.

## 1. Introduction

Curiosity is critical to learning and enables innovative behaviour and efficient information gathering [1–8]. Curiosity has deep phylogenetic and developmental roots in primates, and allows an individual to improve prediction, reduce uncertainty and gain increased control on their environment [2–4,9,10]. Here we operate with a comprehensive definition of curiosity—as a drive to gain information—although some theorists maintain that curiosity is additionally characterized by the absence of instrumental incentives [11]. Lemurs, monkeys and nonhuman great apes (hereafter great apes), our closest living phylogenetic relatives, demonstrate information-seeking behaviours on a number of different tasks. Ringtail lemurs (*Lemur catta*), macaques (*Macaca mulatta*), capuchins (*Cebus apella*), orangutans (*Pongo abelii*), gorillas (*Gorilla gorilla*), chimpanzees (*Pan troglodytes*), bonobos (*Pan paniscus*) and humans (*Homo sapiens*) all seek information to determine the location of hidden food rewards, and optimize their information-seeking behaviours based on the nature of the task [10,12–20].

Great apes also demonstrate curiosity-driven behaviours that improve learning [1,6,9,21]. For example, orangutans who demonstrate higher levels of curiosity are more likely to learn to succeed on problem-solving tasks [22]. Great ape curiosity for physical objects is heightened in younger individuals, increases when more conspecifics are present (due to social and/or response facilitation) and enhances problem-solving skills [6,21–25]. However, a recent study [26] also revealed potential differences between human and chimpanzee curiosity—children were more curious about uncertain options (hidden rewards whose value is unknown) than were chimpanzees, suggesting that children may be more motivated to reduce uncertainty than are chimpanzees. Children’s ability to use curiosity to guide learning and reduce uncertainty emerges early in human ontogeny.

Preverbal human infants produce curiosity-driven behaviours that structure their own cognitive development [3,27–30]. Infants are born with the capacity to guide their attention towards salient, novel and important features in their environment such as areas of high contrast to detect objects, and motion emergence to detect animacy. Infants also bias their attention towards objects and sequences that provide intermediate rates of information (i.e. not too highly surprising nor predictable), to help maximize their rate of learning [3,29,31,32]. Children also intrinsically organize their play behaviour to optimally reduce uncertainty, discover causal structure, and increase information gain [33–41]. Thus, children’s curiosity about objects strategically guides their information-seeking behaviour to maximize learning. The development of curiosity-driven behaviours in chimpanzees is currently underexplored, as access to a large sample of young chimpanzees is notoriously difficult. However, young orangutans demonstrate higher exploratory and approach behaviours towards novel objects than do adults, which suggests that juvenile nonhuman great apes may also utilize curiosity-driven behaviours to structure their own cognitive development [6].

### (a) Social curiosity is adaptive

Although research on curiosity has recently increased, the majority of this research has focused on curiosity about the physical environment—with a paucity of research exploring curiosity about the social environment. Social curiosity, defined here as the drive to gain information about others—including how they think, behave and feel—is a distinct dimension of curiosity [42–44]. Social curiosity is adaptive, enhances the acquisition of evolutionarily relevant social information related to survival and fitness, guides decision-making, and supports the formation and maintenance of social bonds [3,6,9,45]. It also aids with tracking third-party information to form and maintain social relationships [46–51]. Social curiosity is crucial for important partner choice decisions—by gaining information about individuals and how they interact with others, agents can determine which potential partners may be prosocial, competitive, knowledgeable, dominant, harmful, or cooperative [52–54]. For example, it is important for infants to identify potential caregivers and positive social partners [55,56]. Human and nonhuman primates also display heightened responses towards threatening and potentially harmful partners [57,58]. It may be particularly advantageous to display increased attention towards negative social partners and interactions because they are evolutionarily relevant fear stimuli [57,58]. Social curiosity can also be costly, exposing the individual to hostile interactions and requiring time-intensive exploration [59–61]. Thus, mechanisms that optimize social curiosity may be key to the evolution and development of cooperation and greater rates of social tolerance in humans and other apes [62].

While social curiosity has rarely been studied in nonhuman primates and human children, a potential precursor of social curiosity has been investigated in both: biased attention towards faces. Human infants innately direct their attention towards conspecific faces, which provides opportunities to gain social information [63,64]. Chimpanzees and bonobos also preferentially attend towards familiar faces, including those of current groupmates and familiar individuals whom they have not seen for years [65,66]. Initially, human infants preferentially attend towards images of conspecific dyads where the actors are facing away from each other. However, as they develop, children become more like human adults and begin to preferentially attend to facing and interacting dyad images and videos [67,68]. Juvenile macaque monkeys similarly demonstrate a preference for images of facing dyads [67].

The current pre-registered studies were designed to create the foundation for understanding the developmental and evolutionary patterns of human and nonhuman great ape curiosity about social interactions. By developing similar paradigms to test social curiosity in human children and chimpanzees, we aimed to elucidate commonalities and differences in patterns of social curiosity between these species and across human ontogeny. To test whether chimpanzees and children are curious about social interactions and whether social information is rewarding, we designed a simple set-up using two ‘curiosity boxes’. A tablet was placed inside each box, which allowed the experimenter to simultaneously present participants with two different videos. In Experiment 1, participants were presented with a video of social interactions between conspecifics in one box, and a video of a conspecific acting alone in the other box. In Experiment 2, participants were presented with a reward-dispenser that periodically dispensed rewards (marbles for children, and jackfruit seeds for chimpanzees) in one box, and a video of a social interaction in the other box. In Experiment 3, participants were presented with videos of positive social interactions (playing, grooming) in one box, and videos of negative social interactions (conflict) in the other box. Participants were given a short amount of time to open and explore both boxes, and we recorded the length of time they spent watching each video or collecting the rewards. Using this novel design with the curiosity boxes, we were able to compare social curiosity in children and chimpanzees and test the following hypotheses: **Experiment 1:** Children and chimpanzees are more willing to gain information about social interactions than to gain information about an agent acting alone. **Experiment 2:** Social information is rewarding for children and chimpanzees, and they are willing to forgo a reward in favour of social information. **Experiment 3:** Children and chimpanzees are more willing to gain information about negative social interactions than about positive social interactions.

## 2. Methods

### (a) Experiment 1

The main aim of Experiment 1 was to determine whether chimpanzees and children are more willing to gain information about social interactions between two individuals as compared to an individual acting alone.

(i) Chimpanzee Experiment 1

##### Chimpanzee participants

Twenty-seven chimpanzees (13 females, 17–40 years old) living at the Ngamba Island Chimpanzee Sanctuary, Uganda, participated in Experiment 1 (see electronic supplementary material, table S1 for additional information). Chimpanzees have access to a large outdoor forest throughout the day and receive four regular daily feedings, enrichment, water ad libitum and have access to naturally growing plants in their forested island enclosure. Participants voluntarily enter a covered enclosure in the evening (or can choose to stay in the outdoor forest overnight), and voluntarily participate in research in the morning before returning to the outdoor forest. Participants were never deprived of food or water. Testing for all experiments took place in January and February 2024.

##### Materials and stimuli

Two identical wooden boxes (L: 76 cm, W: 31 cm, H: 31 cm) that could be opened via sliding vertical doors with wooden handles were presented on two tables (L: 76 cm, W: 39 cm, H: 60 cm) spaced roughly 150 cm and equidistant (approx. 183 cm) from the starting place of the participant (see electronic supplementary material, figures S1 and S3A). Both boxes contained a Samsung tablet (Samsung Galaxy Tablet A7, Samsung, South Korea) that played videos. The social box played videos of social interactions between two familiar chimpanzees. The nonsocial box played videos of a single chimpanzee acting alone. All videos were filmed at Ngamba Island Chimpanzee Sanctuary, and the original audio was replaced with ambient forest sounds. The nonsocial videos were versions of the social interaction videos (including audio), edited so that only one chimpanzee appeared on the screen and appeared to be acting alone. The videos of the social interactions contained one context each: playing, grooming, conflict and caregiving. The test videos were sex-matched to the participants, such that females saw videos of females, and males saw videos of males.

##### Procedure and design

Each participant participated in two familiarization trials followed by four test trials. Familiarization and the first two test trials occurred on one day, back-to-back. The second two test trials occurred 48 h after the first two test trials (unless participants could not participate until a later time). The order of the test trials, the locations of the Social box and Nonsocial box, and which box was presented first were counterbalanced across participants.

In the familiarization trials, the experimenter (E) placed one tablet in one box and allowed the participant to explore the box for 3 s. E then repeated this familiarization procedure with the other tablet in the other box. To pass the familiarization trials, participants had to open each box and watch each video for 3 s. Chimpanzees were not trained to open the boxes—they were motivated and able to open the boxes the first time they encountered them (see the electronic supplementary material for more details).

##### Test trials

On every test trial, E first called the participant’s name and then held the tablet and started the test video so that the participant could see the content of the video. If this was the social box, the video showed a social interaction between two chimpanzees. If it was the nonsocial box, the video showed a single chimpanzee acting alone. E placed the tablet in the first box and then closed it, and then held the other tablet and started the test video so that the participant could see the content of the video. This video was of the opposite condition (social versus nonsocial) from the first video, and of a different context (playing, grooming, conflict and caregiving). E placed the tablet in the second box and closed it and said ‘Ready’ to the caretaker assistant, who then opened the door to the testing room and allowed the participant to enter. E held peanuts in one hand up to the bars (in the middle and equidistant to both boxes) and fed them to the participant to centre them between the boxes. E then stepped back, stood roughly 90 cm behind the boxes, and looked down at the ground. The participant was given 1 min to explore both boxes. After 1 min, the caretaker assistant called the participant back to the enclosure adjacent to the testing room. E repeated this procedure for Trial 2. In each test trial, a different context was shown in the social and nonsocial boxes. The video context in each box varied across the four trials, such that participants saw one video from each of the four contexts in both the social and nonsocial boxes.

##### Data scoring and analyses

We calculated the total time the participant looked in each box, beginning when participants began raising the door to the box and ending when the box was fully shut again. If participants raised the door to a box multiple times in a single trial, their total looking time across the trial was summed. Coding was executed by raters who were blind to the trial and video conditions, and inter-rater reliability analyses produced good to excellent kappa values between 0.78–0.96. In all models for each experiment we analyse the raw looking times to each box. We fitted simple linear mixed-effects models using the *lmer* function from the lme4 package [69] using the statistical software R (version 2023.12.1). In Model 1, we tested the prediction that chimpanzees would spend more time watching the videos of the social interactions between two chimpanzees as compared to the videos of the single chimpanzee. We used a dependent variable of looking time in each box, with an interaction between condition (social or nonsocial) and sex as the test effect; social context and nonsocial context as fixed effects; and random slopes for trial number within participant ID as well as random slopes for participant ID. In Model 2, we tested the prediction that chimpanzees would be likelier to open the social box as compared with the nonsocial box, measured by whether they opened the box in each trial with a binomial error structure (1 = opened box, 0 = did not open box). We used a dependent variable of whether the chimpanzee opened the box, with an interaction between condition (social or nonsocial) and sex as the test effect; social context, and nonsocial context as the fixed effects; and random slopes for trial number within participant ID as well as random slopes for participant ID. Finally in an exploratory Model 3, we tested whether participants were more likely to open the social or nonsocial box first in each trial. We used first box opened in a trial as the dependent variable, Z-transformed trial number as a fixed effect, and random slopes for trial number within participant ID as well as random slopes for participant ID. Across all studies, we used a significance threshold of 0.05 when reporting *p*-values, and reported trends between 0.05 and 0.1 to avoid arbitrary dichotomization of significance [70]. We also report conditional *R*² as an effect size measure for all mixed-effects models to quantify the amount of variance explained by the fixed and random effects in each model (with an *R*² value of 0.1 indicating a small amount and a value of 0.5 indicating a large amount of variance explained by the model). However, uncertainty measures for *R*² values are not available and thus they should be interpreted cautiously [71].

##### Results

**Figure 1.**
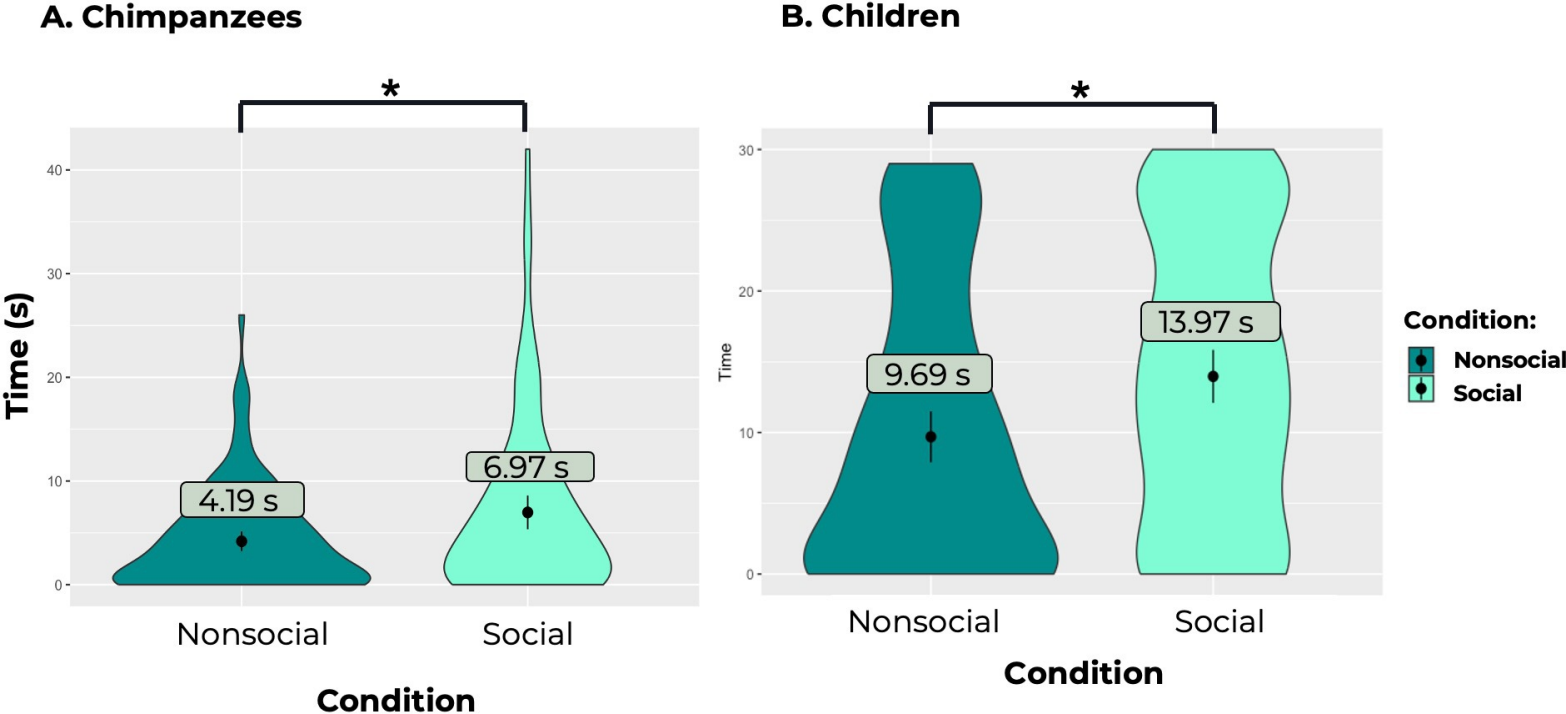
Amount of time (in seconds) that (A) chimpanzees and (B) children spent watching the social interactions (the social box; light green) versus the single conspecific acting alone (the nonsocial box; dark green). Chimpanzees and children spent significantly more time looking at the videos of the social interactions as compared to videos of the single conspecific acting alone. Note that trials for chimpanzees lasted 60 s, while trials for children lasted 30 s. Points denote looking time means in each condition, and lines denote ±1 standard deviation.

Chimpanzees spent significantly more time watching the videos of social interactions (x̄ = 6.972 ± 8.534 (SD)) than the videos of chimpanzees acting alone (x̄ = 4.185 ± 4.932 (SD); estimate = 2.787 ± 0.833 (SE); *p* = 0.001, see figure 1A). There was no significant effect of sex (estimate = 0.641 ± 1.495 (SE), *p* = 0.672). The effect of trial number trended towards significance (estimate = −0.947 ± 0.501 (SE), *p* = 0.065), indicating that chimpanzees watched the videos for shorter amounts of time as the trials progressed. Model 1, which included the test effect of condition (social or nonsocial), was significantly better than the reduced Model 1 without this effect (*p* = 0.0008; *R*² = 0.297). Chimpanzees were as likely to open the Social as the Nonsocial Box in a given trial (Model 2, as measured by whether they opened each box in each trial; estimate = 0.056 ± 0.063 (SE), *p* = 0.382). The effect of trial number again trended towards significance in Model 2 (estimate = −0.057 ± 0.032 (SE), *p* = 0.083), suggesting that chimpanzees were less likely to open the boxes as the trials progressed. Model 2 with the test effect of condition (social or nonsocial) was not significantly better than the reduced Model 2 without this effect (*p* = 0.369; *R*² = 0.315). As trials progressed, chimpanzees became more likely to open the social box first (estimate = 0.780 ± 0.2903 (SE), *p* = 0.003).

### (ii) Child Experiment 1

#### Child participants

Thirty-five 4−6-year-old children (15 females, mean 66 months) living in the San Francisco Bay Area participated in this study. For all studies, children were tested either at the Lawrence Hall of Science in Berkeley or at the Oakland Zoo in Oakland, California. Children were recruited directly at these sites, and participated only once they had provided verbal assent and their legal guardians had signed an IRB-approved permission form. Children only participated in one out of the three experiments, and received a small sticker for their participation. Data collection occurred June through December 2023. Five children did not correctly complete the study due to experimenter error and therefore were not included in the final analyses. Thus thirty children total completed this study.

The materials, stimuli, procedure, design and analyses were nearly identical to those of Chimpanzee Experiment 1 (see electronic supplementary material, figures S2 and S3B). The only difference was that children had 30 s to explore the boxes (to keep testing time to a minimum), and we explored the developmental patterns of curiosity (see the electronic supplementary material for more details).

#### Results

Children spent significantly more time watching the videos of social interactions (x̄ = 13.966 ± 10.339 (SD)) than the videos of children acting alone (x̄ = 9.697 ± 9.902 (SD); estimate = 4.213 ± 1.934 (SE), *p* = 0.038; see figure 1B). Model 1 with the test effect of condition (social or nonsocial) was significantly better than the reduced Model 1 without this effect (*p* = 0.002; *R*² = 0.198). Children were more willing to open the box with the videos of the social interactions compared with the box with the videos of the single child acting alone (as measured by whether they opened each box in each trial; estimate = 0.134 ± 0.065 (SE), *p* = 0.048). Model 2 with the test effect of condition (social or nonsocial) was also significantly better than the reduced Model 2 without this effect (*p* = 0.043; *R*² = 0.222). Children also trended towards opening the box with the videos of the social interactions wider (x̄ = 6.815 ± 3.778 (SD)) than they opened the box with the videos of the single child acting alone (x̄ = 5.815 ± 4.426 (SD); as measured by the maximum height they lifted the box door in each trial; estimate = 0.997 ± 0.580 (SE), *p* = 0.096). Model 3 with the test effect of condition (social or nonsocial) trended towards being significantly better than the reduced Model 3 without this effect (*p* = 0.088; *R*² = 0.251). Children were not significantly more likely to open one box before the other across trials (estimate = 0.2238 ± 0.2942 (SE), *p* = 0.4387).

### (b) Experiment 2

The main aim of Experiment 2 was to determine whether social curiosity is rewarding for chimpanzees and children. Specifically, here we test whether there is a trade-off between social rewards and food rewards, measured by whether participants are willing to forgo a reward to gain social information.

#### (i) Chimpanzee Experiment 2

##### Chimpanzee Participants

Sixteen chimpanzees (8 females, 17–40 years old) living at the Ngamba Island Chimpanzee Sanctuary, Uganda, participated in Experiment 2. All chimpanzees in Experiment 2 also participated in Experiment 1.

##### Materials and stimuli

All materials were the same as in Experiment 1. The only difference was that we showed videos in the tablet box, and the other box contained a remote-controlled reward dispenser (Petsafe Train n’ Praise Treat Dispenser, USA) that would dispense one to two jackfruit seeds (carved into round shapes) when E pressed the remote-control (the reward box).E would inconspicuously dispense the jackfruit seeds every 15 s after the trial started (for a total of four times across the 1 min test trials). The videos contained different scenes of conflicts between familiar chimpanzees (conflict scenes were different from those in Experiment 1 and contained multiple adult males and females). The original audio was used for all videos, such that participants heard the sounds of the conflicts when the videos were playing. In the no-information condition, videos did not play and the tablet was left blank. This was to ensure that there was a clear difference between the social and no-information conditions, as at the time it was unknown whether chimpanzees differentiated between videos of social interactions versus videos of a single conspecific acting alone.

##### Procedure and design

The trial procedure, design and analyses were similar to that of Chimpanzee Experiment 1. Each participant first participated in a food preference test to ensure that they were motivated to eat jackfruit seeds (with 13/16 chimps eating all 8 jackfruit seeds), and then two familiarization trials followed by four test trials (two per condition; see the electronic supplementary material for more details).

##### Data scoring and analyses

In Experiment 2, we tested whether social information is rewarding for chimpanzees, and whether they are more willing to gain social information over a reward. Therefore, we calculated the total time they spent *away* from the reward box, as well as the total time they spent at the tablet box. We calculated time away from the reward box as total trial time (60 s) minus time at the reward box (beginning at the time at which participants began raising the door to the reward box, and ending when it was fully shut again). Thus in Model 1, we test whether chimpanzees spend more time away from the reward in the social condition as compared with the no-information condition, and in Model 2 we test whether chimpanzees spend more time at the tablet box in the social versus no-information condition in order to determine whether social information is so rewarding for chimpanzees that they are willing to give up a reward in order to gain social information. Exploratory Model 3 tested whether participants were more likely to open the social or nonsocial box first in each trial.

##### Results

**Figure 2.**
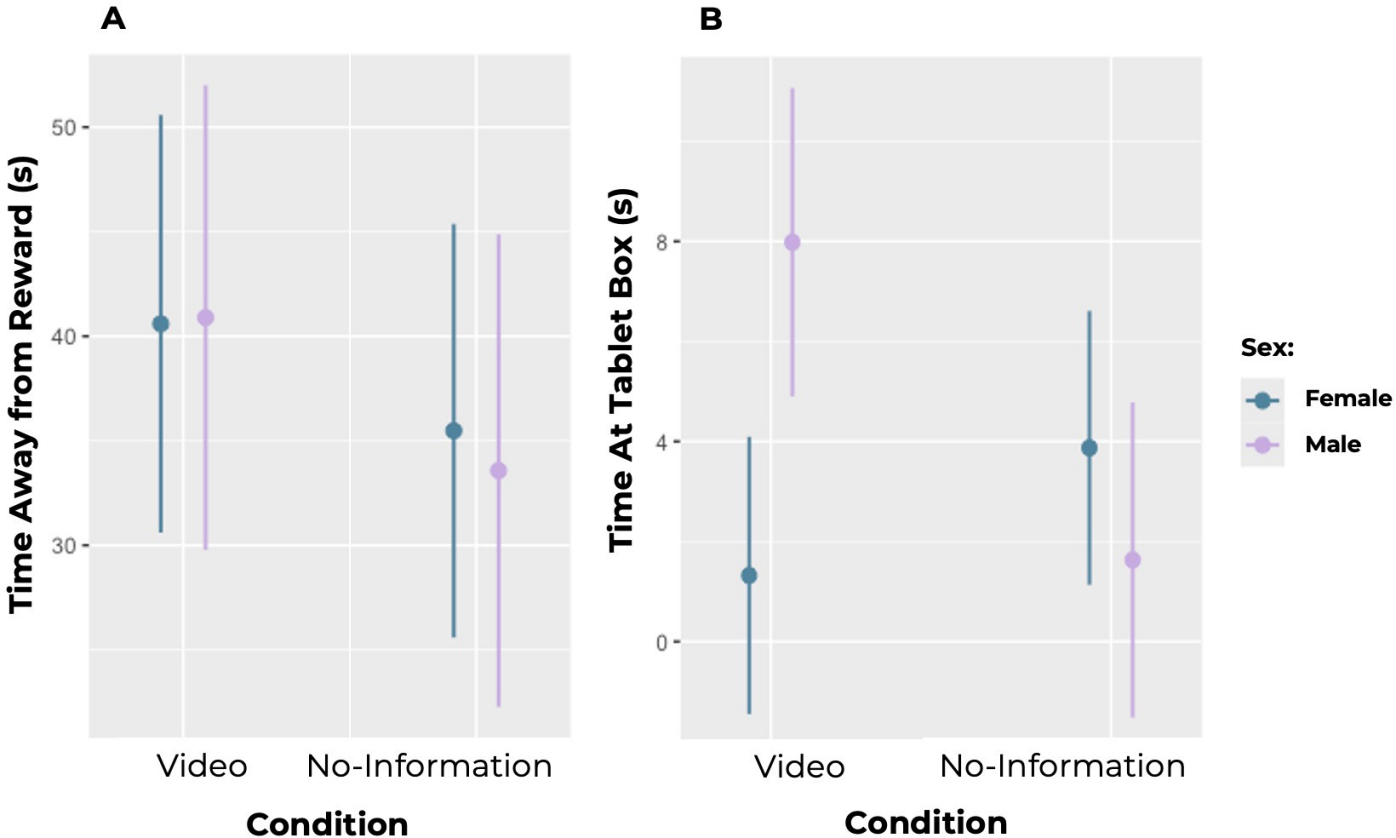
(A) Predicted amount of time (in seconds) that chimpanzees spent away from the reward box, by sex. Chimpanzees spent significantly more time away from the reward box in the video condition as compared to the no-information condition. Points denote means and lines denote 95% confidence intervals. (B) Predicted amount of time (in seconds) that chimpanzees spent at the tablet box, by sex. Male chimpanzees spent significantly more time at the tablet box in the video condition as compared with the no-information condition, while female chimpanzees trended towards spending more time at the tablet box in the no-information condition. Points denote means and lines denote 95% confidence intervals.

To examine whether chimpanzees were willing to give up a reward to gain social information, we compared how long chimpanzees spent *away* from the reward box in each trial based on the condition (video versus no-information, Model 1). We found a significant effect of condition such that chimpanzees spent significantly more time away from the reward box in the video condition (x̄ = 40.281 ± 18.006 (SD)) than in the no-information condition (x̄ = 35.094 ± 15.272 (SD); estimate = 6.108 ± 2.925 (SE), *p* = 0.0483) (see figure 2A). The model with the interaction between the test effects of condition and sex was not better than the model with condition and sex as separate test effects (*p* = 0.747) and therefore we dropped this interaction. The model with these as separate test effects was significantly better than the reduced model without the effect of condition (*p* = 0.039; *R*² = 0.585).

To examine whether chimpanzees were more willing to gain social information in the video condition (Model 2), we analysed how long chimpanzees spent at the tablet box in each condition (video or no-information). We found a significant effect of the interaction between condition and sex (estimate = −8.901 ± 3.062 (SE), *p* = 0.007). The model with the interaction between these test effects was better than the reduced model without this interaction (*p* = 0.011; *R*² = 0.356). Therefore, we decided to inspect the data from female and male chimpanzees separately using exploratory models. Upon further inspection, we found that male chimpanzees spent significantly more time at the tablet box in the video condition (x̄ = 7.643 ± 5.878 (SD)) than in the no-information condition (x̄ = 1.714 ± 2.335 (SD); estimate = −5.453 ± 1.625 (SE), *p* = 0.003), whereas females trended towards spending less time at the Tablet Box in the video condition (x̄ = 2.278 ± 6.849 (SD)) than in the no-information condition (x̄ = 3.111 ± 5.687 (SD); estimate = 4.085 ± 2.345 (SE), *p* = 0.099) (see figure 2B). As trials progressed, chimpanzees became more likely to open the reward box first and less likely to open the tablet box first (estimate = −0.115 ± 0.0507 (SE), *p* = 0.02683).

### (ii) Child Experiment 2

#### Child participants

Thirty-six 4–6-year-old children (16 females, mean 64 months) participated in this experiment. Four children did not correctly complete the experiment due to experimenter error and were not included in the final analyses. Thus a total of 32 children completed this experiment.

The materials, stimuli, procedure, design and analyses were similar to those of Chimpanzee Experiment 2. The only differences were that children had 30 s to explore the boxes, they received marble rewards rather than jackfruit seeds, they saw a video of a single individual instead of no video (in the nonsocial condition), they saw unfamiliar individuals who were sex-matched to the participants in all four original contexts and we explored the developmental patterns of curiosity. In addition, the audio for each video was replaced with ambient forest sounds. For further details about Child Experiment 2 materials, stimuli, procedure, design and analyses, see the electronic supplementary material.

#### Results

**Figure 3.**
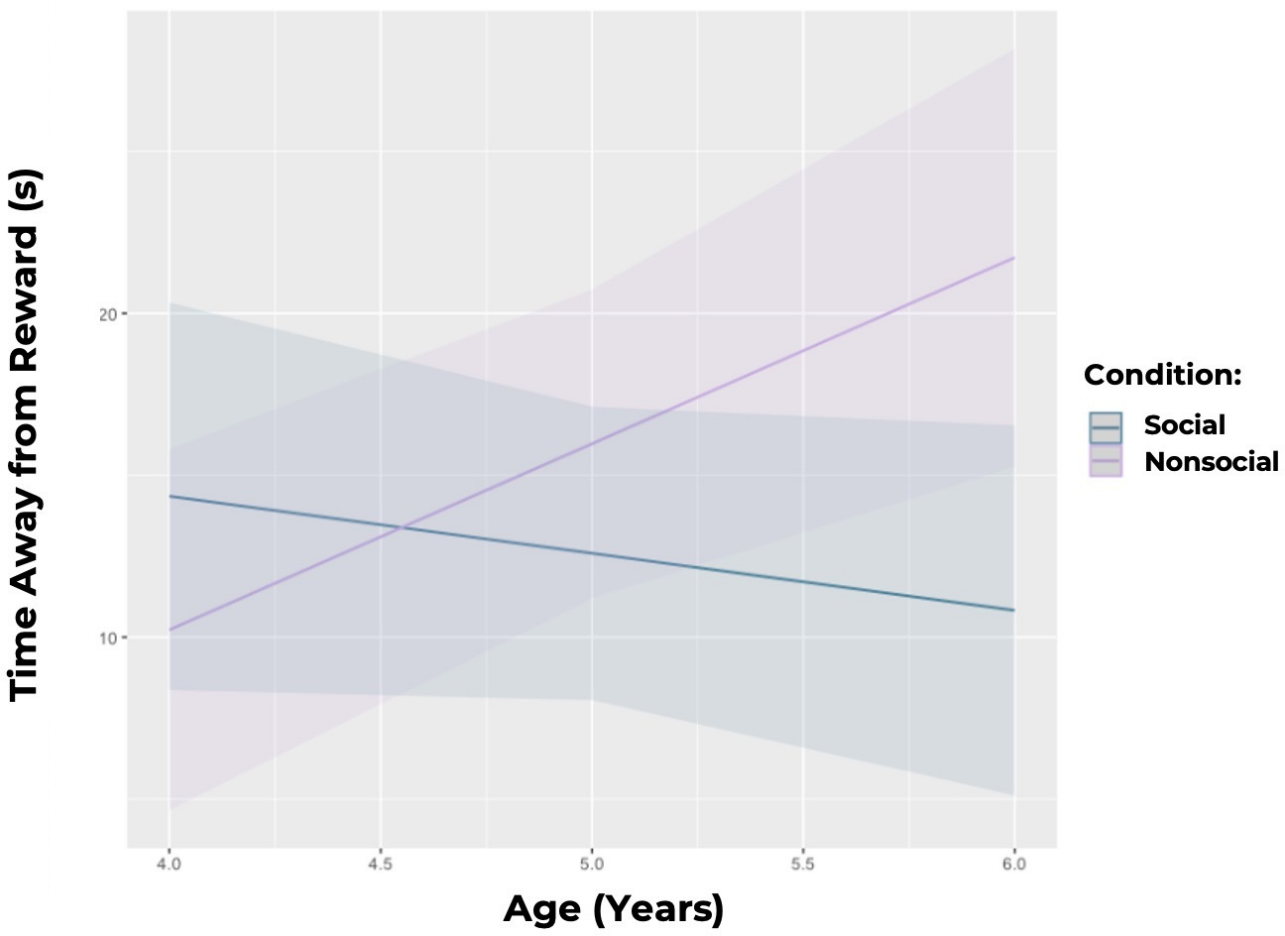
Predicted amount of time (in seconds) that children spent away from the reward box, by age. Younger children (4 year olds) spent significantly more time away from the reward box in the social condition as compared with the nonsocial condition and as compared with older children (5 and 6 year olds). Shaded areas denote 95% confidence intervals.

To examine whether children were more willing to give up a reward to gain social information than they were to gain nonsocial information, we first compared how long children spent *away* from the reward box in each trial based on the condition (social versus nonsocial). We found a significant interaction between condition (social versus nonsocial) and age (estimate = 0.542 ± 0.176 (SE), *p* = 0.003). Specifically, we found that younger children (4 year olds) spent more time away from the reward box in the social condition (x̄ = 11.75 ± 9.244 (SD)) than in the nonsocial condition (x̄ = 7.65 ± 7.583 (SD)), whereas older children (5 and 6 year olds) spent more time away from the reward box in the nonsocial condition (x̄ = 16.10 ± 11.462 (SD)) than in the social condition (x̄ = 10.326 ± 9.884 (SD)). Model 1 with the test effect of condition × age was significantly better than the reduced model without this effect (*p* = 0.006; *R*² = 0.269) (see figure 3). We next analysed the total time children spent at the tablet box in the social versus nonsocial condition, and found a significant interaction between condition and age such that younger kids (4 year olds) spent more time looking at the social videos (x̄ = 4.30 ± 10.229 (SD)), while older kids (5 and 6 year olds) spent more time looking at the nonsocial videos (x̄ = 10.95 ± 11.869 (SD); estimate = 0.387 ± 0.178 (SE), *p* = 0.033). Model 2 with the test effect of condition × age was significantly better than the reduced model without this effect (*p* = 0.026; *R*² = 0.262). Children trended towards being more likely to open the video box first in the nonsocial than in the social condition (estimate = 0.14884 ± 0.07781 (SE), *p* = 0.06557).

### (c) Experiment 3

The main aim of Experiment 3 was to determine whether chimpanzees and children are more willing to gain information about negative social interactions than about positive social interactions.

#### (i) Chimpanzee Experiment 3

##### Chimpanzee participants

Twenty-five chimpanzees (11 females, 17–40 years old) living at the Ngamba Island Chimpanzee Sanctuary, Uganda, participated in Experiment 3.

##### Materials and stimuli

All materials were the same as in Experiment 1. The only difference was that we showed new videos of positive interactions (the positive box), and new videos of negative interactions (the negative box). The positive videos included two contexts: playing and grooming. The negative videos included conflicts. All videos contained multiple familiar adult males and females, and the original audio was replaced with natural forest sounds.

The procedure, design and analyses were nearly identical to those of Chimpanzee Experiment 1 (see the electronic supplementary material for more details).

##### Results

We found that chimpanzees did not spend significantly different amounts of time watching the videos of the positive social interactions (x̄ = 3.796 ± 4.842 (SD)) versus the videos of the negative social interactions (x̄ = 4.449 ± 6.884 (SD); estimate = −0.653 ± 0.819 (SE), *p* = 0.426). Chimpanzees were also not more likely to open the box with the positive social interactions versus the box with the negative social interactions (estimate = 0.010 ± 0.059 (SE), *p* = 0.864). Chimpanzees were not significantly more likely to open one box before the other across trials (estimate = −0.2379 ± 0.2666 (SE), *p* = 0.1354).

### (ii) Child Experiment 3

#### Child participants

Thirty-five 4−6-year-old human children (16 females, mean 64 months) participated in this experiment. Three children did not correctly complete the experiment due to experimenter error and therefore were not included in the final analyses. Thus 32 children total completed this experiment.

The materials, stimuli, procedure, design and analyses were similar to those of Chimpanzee Experiment 3; the only difference was that children had 30 s to explore the boxes, they saw unfamiliar individuals who were sex-matched to the participants, and we explored the developmental patterns of curiosity in children. The positive videos included two contexts: playing and grooming (i.e. doing each others’ hair). The negative videos also included two contexts: conflict (i.e. tug of war over a toy) and crying (i.e. one child crying while another child yells at them). For all videos, the original audio was replaced with ambient forest sounds (see the electronic supplementary material for more details).

#### Results

**Figure 4.**
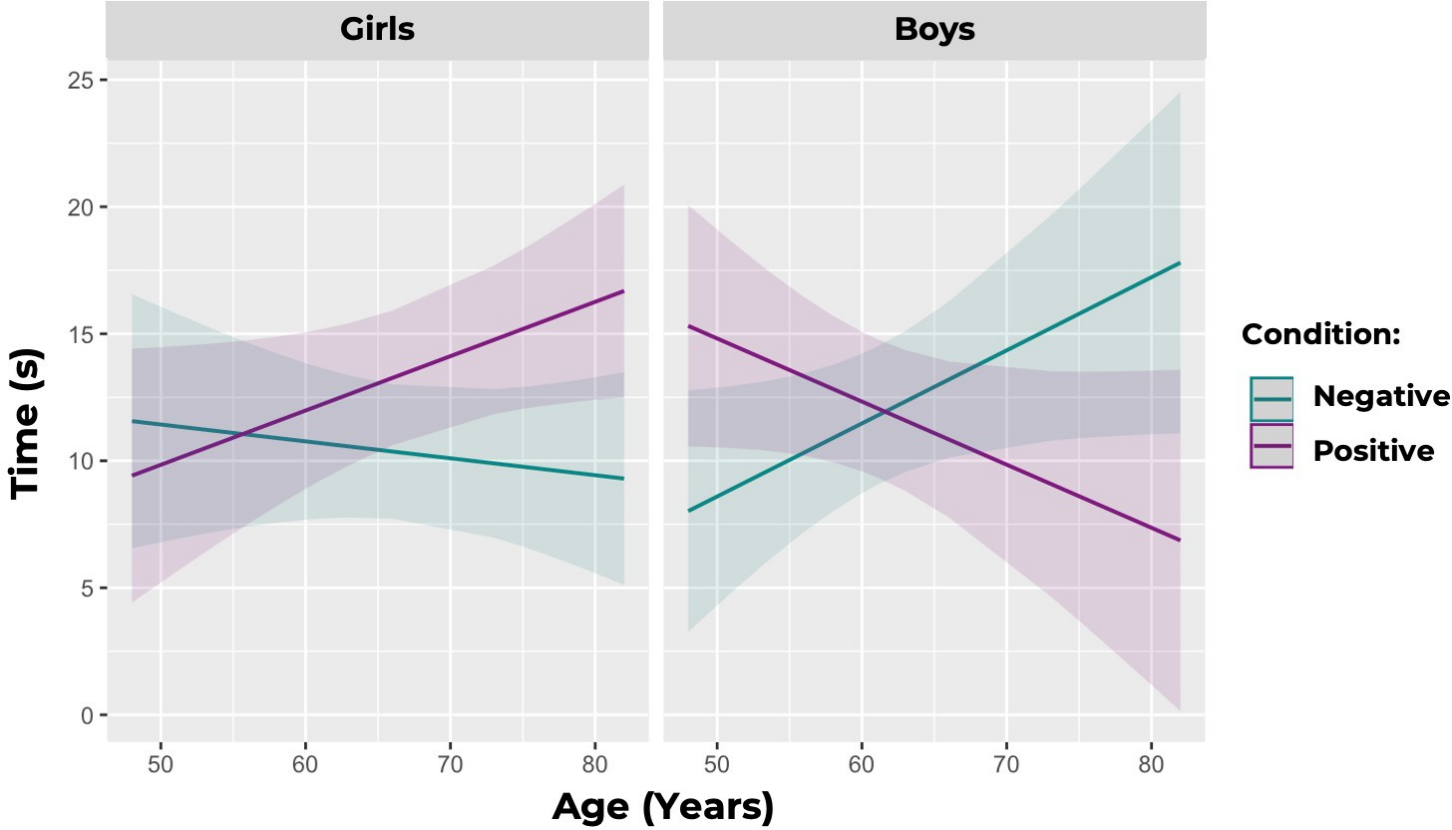
Predicted amount of time (in seconds) that children spent watching the positive interactions (purple) and the negative interactions (teal), by age. Girls trend towards spending more time watching positive interactions, while boys shift towards spending significantly more time watching negative interactions as they age. Shaded areas denote 95% confidence intervals.

We found a significant three-way interaction between condition, age in months, and gender (estimate = −0.8177 ± 0.265 (SE), *p* = 0.002) for Model 1 (looking time to each video). Model 1 with this three-way interaction was significantly better than the model without this interaction (*p* = 0.012; *R*² = 0.053). Therefore, we analysed the data from boys and girls separately. For boys, we found a significant interaction between condition and age such that younger boys (4 year olds) spent more time watching the positive interactions (x̄ = 12.75 ± 12.029 (SD)), while older boys (5 and 6 year olds) spent more time watching the negative interactions (x̄ = 12.688 ± 10.825 (SD); estimate = −0.536 ± 0.223 (SE), *p* = 0.018). For girls, we found a marginally significant interaction between condition and age such that girls trend towards spending more time watching the positive interactions (x̄ = 13.531 ± 10.276 (SD)) than the negative interactions (x̄ = 10.281 ± 10.324 (SD)) as they age (estimate = 0.280 ± 0.152 (SE), *p* = 0.067; see figure 4). We found a significant interaction between condition, age in months and gender (estimate = −0.025 ± 0.011 (SE), *p* = 0.019) for Model 2 (likelihood to open each video). Model 2 with this three-way interaction was marginally significantly better than the model without this interaction (*p* = 0.056; *R*² = 0.183). Therefore, we analysed the data from boys and girls separately. For boys, we found a marginally significant interaction between condition and age such that younger boys are more likely to open the box with the positive social interactions, while older boys are more likely to open the box with the negative social interactions (estimate = −0.017 ± 0.009 (SE), *p* = 0.0529). For girls, we did not find a significant interaction between condition and age (estimate = 0.008 ± 0.006 (SE), *p* = 0.217), and therefore we dropped this interaction and re-ran the model. We found a significant effect of condition, such that girls were more likely to open the box with the videos of the positive interactions (estimate = 0.156 ± 0.007 (SE), *p* = 0.033). Finally, we did not find a significant interaction between condition, age and gender (estimate = −0.130 ± 0.095 (SE), *p* = 0.174) for Model 3 (box door height). Children did not open the door of the positive box significantly higher than the door of the negative box. Girls trended towards opening the positive box first across trials (estimate = 0.7445 ± 0.4356 (SE), *p* = 0.0874), whereas boys were not significantly more likely to open one box before the other across trials (estimate = −0.1539 ± 0.3827 (SE), *p* = 0.2155).

## 3. Discussion

We tested whether chimpanzees and children are more curious about social interactions versus a single agent (Experiment 1), whether they are willing to pay a cost to gain social information (Experiment 2), and whether they are more curious about positive or negative social interactions (Experiment 3). Combined, we found that both chimpanzees and children are socially curious creatures. Chimpanzees and children are more curious about social interactions than the actions of a single conspecific, young children and male chimpanzees are willing to pay a cost to gain social information, and boys become more curious about negative social interactions while girls become more curious about positive social interactions as they develop.

In Experiment 1, chimpanzees and children are significantly more motivated to watch videos of social interactions compared with videos of a conspecific acting alone. Children are also significantly more likely to open the social box compared with the nonsocial box, and trend towards opening the door of the social box wider. What is driving this effect? One option is that chimpanzees and children possess a specific drive to gain information about social interactions. Another option is information quantity: a social interaction between two agents naturally contains more information than does a single agent acting alone, as the interaction provides information not only about the two actors but also about the nature of their relationship. Therefore, children and chimpanzees might show stronger curiosity for social interactions compared with individual agents to gain more information. Previous research has shown that children and chimpanzees discriminate between different sources of information and adaptively maximize their information gain and rates of learning [3,29,31,32]. Future research should tease apart these options.

In Experiment 2, chimpanzees and younger children spend significantly longer amounts of time away from the reward box when there is social information to be gained in the tablet box. In addition, younger children and male chimpanzees spend more time at the tablet box in the social condition compared with the nonsocial or no-information condition, respectively, and thus are more willing to gain social information than are older children or female chimpanzees, respectively. The combined measures of time away from the reward box and time at the tablet box reveal that younger children and male chimpanzees are probably willing to ‘pay for gossip’, or abandon opportunities to gain rewards in order to gain social information. It may be that social information is especially important and rewarding for younger children and male chimpanzees. However, as the no-information box in chimpanzee Experiment 2 did not contain any videos or sounds, it could be that male chimpanzees preferred to approach the auditory information and watch any social videos over gaining a reward. As the video stimuli for chimpanzees included conflicts between conspecifics, it may also be that auditory and visual information about social conflicts is especially rewarding and important for male chimpanzees as compared with females, perhaps because males are more likely to be involved in conflicts (initiating about 98% of all conflicts by some measures) [72]. Female chimpanzees typically avoid direct physical competition and demonstrate low rates of aggression, which may explain why they might avoid directly watching these videos.

In Experiment 3, girls trend towards being more curious about positive social interactions whereas boys become significantly more curious about negative social interactions as they age. Chimpanzees do not demonstrate any significant difference in curiosity between positive and negative interactions. Children probably experience differences in social curiosity towards positive and negative social interactions that are linked to demographic differences in age and gender. These differences in social curiosity are probably a product of a combination of differing socialization patterns and evolutionary pressures that boys and girls have faced throughout human evolution [73,74]. For example, boys and men may have faced more selective pressures to attend to and engage in competitive, physical conflicts and coalitions than girls and women faced [73]. A meta-analysis also found a significant difference in how parents encourage sex-typed activities and sex-stereotyped characteristics with their sons versus their daughters [74]. Future research could explore the disparate contributions of selective pressures and socialization patterns on social curiosity in human children.

### (a) Limitations and future directions

This research, to our knowledge, represents the first cross-species comparative tests of social curiosity. However, there are some limitations and areas of follow-up research that could be informative. First, children are typically more familiar with observing screens than are chimpanzees; this may explain why chimpanzees watched the videos for shorter amounts of time than did children [75]. A second limitation concerns our ability to give instructions to children but not to chimpanzees. The instructions to child participants included the phrase: ‘You can open either this box or that box. It’s up to you.’ This could have potentially inflated the time children spent looking at the boxes. We could have instead instructed children they ‘could open this box, that box or neither—it’s up to you’ to make the procedure more comparable across species. Children saw videos of unfamiliar individuals, whereas chimpanzees saw videos of familiar conspecifics. This could have led to greater curiosity in children because unfamiliar individuals convey more novel information than do familiar individuals. We compared human children with adult chimpanzees, which could produce issues when generalizing comparative results between species. Future research with human adults could generate more informative comparisons with adult chimpanzees. Finally, developmental patterns of social curiosity in chimpanzees could not be explored in the present experiments due to a lack of access to a large sample of young chimpanzees. With the creation of multi-institution collaborative networks such as ManyPrimates [76] and the Primate Eye Tracking Network, it may be possible to explore developmental patterns of social curiosity by including a larger sample of juvenile nonhuman great apes.

As our results indicate some clear developmental patterns in social curiosity, further exploration of these patterns could include a larger sample of children from a broader age range and different cultures. Investigating social curiosity in other great apes like bonobos, orangutans and gorillas could further elucidate evolutionary patterns that may shape curiosity in our great ape family. More social populations of orangutans have higher exploration tendencies, and exploration seems to be socially induced in these species [6,77,78]. These patterns led Forss and colleagues to propose the ‘social curiosity hypothesis’—that highly social great ape species (chimpanzees and bonobos) are less curious about physical objects and their environment compared with less social great ape species (orangutans) when they are tested in isolation [21,59,77]. Thus, another worthwhile future direction could be to probe the effects of sociality on social curiosity in great apes. Finally, we did not explore whether specific social relationships related to relative dominance status, kinship or friendship influenced social curiosity in children and chimpanzees. An exciting future approach could systematically vary these factors to determine their potential influences on social curiosity.

## 4. Conclusion

Chimpanzees and children find social information rewarding and demonstrate cognitive capacities to discriminate and pursue settings that provide a maximal amount of social information. These abilities are likely to be adaptive, as they amplify the acquisition of evolutionarily relevant social information, guide decision-making, and underpin the formation and maintenance of social bonds [3,6,9,45]. Social curiosity may also aid decision-making processes linked to partner choice by enabling successful tracking of third-party relationships in order to use third-party information to cultivate strong social relationships [46,47,49–51,79–81]. Social curiosity therefore probably has deep evolutionary and developmental roots in great apes that have supported the evolution of humans’ cooperative, ultra-social communities.

## Data Availability

The data, analysis code and example stimuli associated with this manuscript are available on Dryad and Zenodo [82,83]. Supplementary material is available online [84].
